# Discussions at the Thirty-Sixth Annual Meeting of the Mississippi Valley Dental Association

**Published:** 1880-04

**Authors:** 


### DISCUSSIONS AT THE THIRTY-SIXTH ANNUAL MEETING OF THE MISSISSIPPI VALLEY DENTAL ASSOCIATION.

The first question discussed was, "Treatment and Management of Exposed Tooth Pulps.

Dr. J. Taft opened the discussion as follows: I scarcely know whether it is best to devote much time to this subject or not; a great deal of time has been given to it at almost every previous meeting; yet we do not know as much about it as we should, for the welfare of our patients; this is shown to be true, when some of our very best men in the profession to whom we are inclined to look for instruction, say they frequently fail, and assert that they cannot attain success in treatment of the exposed pulps of teeth; that they occasionally find a case amenable to treatment, but a large proportion of cases cannot be treated successfully. The faulty practice grows out of imperfect views. There are various phases of diseased tooth pulp. This particular structure has not been studied as it ought to be. Dr. Boedieker of New York, raised the question at the last meeting of the American Dental Association, as to whether there were vessels in the pulps of teeth. Dr. Atkinson followed him and said it had never been demonstrated. If this is true, if the men who should know are in this position, it certainly shows that there is occasion for investigation on the subject; more should be known of this matter, for the pulp is a very important part of the teeth; it performs a very essential work for the teeth before their completion, and undoubtedly it is the main support after the completion; from it the life of the dentine is derived, for when this structure is devitalized or removed from the teeth, life in the dentine no longer remains and the teeth begin to deteriorate and the integrity of the structure is sooner or later destroyed and the teeth soon break away, this fact emphasises to us the importance of this portion of the teeth.

Another very important fact is, that in many cases, soon after the devitalization of the pulp occurs there is more or less of irritation about the teeth; they decay and poisonous matter is taken into the living tissue and irritation occurs in such teeth which is not found while the pulp is living. The ordinary irritants that are present in the mouth do not effect the pulp until it is exposed; some pulps do indeed become devitalized before being exposed, but it is not usually irritated to a marked degree before exposure; then some pulps will live for years after exposure. It greatly depends on the subject and the condition of the mouth and the character of the disease and agent that produces the disease, and besides the general system has great influence in this matter; on it the susceptibility of the tooth pulp to disease finds a corresponding susceptibility. The pulp is much more liable to disease in early life than in the mature years, teeth are, as a rule, more dense later in life; then at different periods there will be a difference in the degree of susceptibility to disease, the pulp and tissue of the teeth are found to be susceptible to all the vicissitudes and changes that may take place in the general system.

In treating exposed pulps, systemic treatment cannot well be overlooked, it will be well to tone up the patient and bring about a better state of health by better food and nutrition, then, in local treatment remove all irritants possible, and if there are some that remain, neutralize them with the proper disinfecting agents. In some localitie s miasmatic influence will very much modify the facility of pulp treatment. Dr. Rehwinkel has often said, that in the

Scioto Valley it was almost impossible to treat diseased pulps with any hope of success. In swampy and malarious districts it is more difficult to attain success in treatment, in other places success is easily attained. If the pulp has been simply exposed by improper manipulation it can be closed up at once. There is nothing to be gained in a case of this kind by either local or systemic treatment; the proper method if you have exposed it, is to close it as soon as possible and the desired end will be gained, and in cases where there is only slight irritation, they may be treated and closed at once.

Dr. King of Pittsburgh, gave us the idea of placing over the exposed tissue a paste composed of oxide of zinc and carbolic acid. In the use of this, he remarks that he has had almost universal success, in cases even where there is active inflammation. I do not regard such treatment simply, as practicable, when the inflammation has been of short duration and is going rapidly forward, depletion may be employed and the inflammation relieved in this way. Systemic treatment may be employed to great advantage, the circulation must be reduced in the part. I have frequently used leeches on the gums with good effect; there is no danger in cutting the pulp; it may sometimes be half cut away and the remainder will live, and after the blood is drawn away it may be covered with a proper material. Carbolic acid and chloride of zinc paste is much used in pulp treatment, but it does not meet the requirements and, sometimes increases the inflammation. The proper local treatment consists in the removal of irritants, relieving the excessive circulation in the part and then gentle stimulation. The surface of the pulp is sometimes found covered with dead or partially dead tissue, this may readily be removed by the application of a paste of pipsin, this should remain for from 24 to 30 hours and then be removed, it leaves the pulp entirely freed from its *debris*, the pulp will regain a healthy state: at the same time we must take into account the systemic condition and the surrounding circumstances, controlling and managing all these to the best interests of the patient; when the pulp has been restored to health it can be closed up—covered over so there will be no pressure upon it; this can be easily done by the use of the proper material. Formerly gold, lead, horn, wood, &c., were used and frequently the treatment with these would not be successful. Dr. Harris remarks in the earlier editions of his work that often after the use of those materials, though they would succeed sometimes for years, disease would occur. The difficulty was to find a substance that would occupy all the space and yet cause no pressure on the pulp, for when the pulp is pressed by the covering, disease is liable to occur.

Various non-irritant materials have been devised that become firm at once, fill all the space and yet make no pressure. Some of these contain lactic acid and that is good, some use carbolic acid still, but there are other things better, I have sometimes made a preparation with water or glycerine and thus avoided the use of carbolic acid; but the non-irritant preparations now made are all sufficient in my opinion for the purpose.

*Dr. Hunter*: I agree with Dr. Taft to a certain extent, but not in the idea of cutting away a portion of the pulp and retaining the rest, then, again, my sense of touch is not acute enough to know when applying the covering just when the material has filled all the space and yet have no pressure on the tooth, how shall we apply such a preparation to the pulp of an upper molar.

*Dr. Taft*: You must have the preparation so it will flow into the orifice of exposure, you can afterwards remove the excess of fluid by using bibulous paper, if this is done well, you have a covering firm enough to receive a gold filling. As to removing part of the pulp, I have seen a number of cases where the tooth has been so treated and the remainder of the pulp still living, the chamber had been filled up; I think it always desirable to save the pulp tissue in the root of a tooth if possible, a great deal depends on having a fine pointed instrument to work with in filling up the chamber where a part of the pulp has been removed. I do not use arsenous acid to devitalize teeth, I think it is not safe.

*Dr. Keely*: I never devitalize a tooth without first making an effort to save its life, for, I know wherever there is devitalization the teeth begin to decay.

*Dr. Jamison*: I have had great success in treating exposed tooth pulps where the tooth had been aching but for a short time, by making an application of creosote and oxide of zinc, and after this I have no hesitancy in cleaning and filling the cavity immediately. I have tried both ways, but I have greater success when I complete the operation at one sitting, than if I wait, I never use an amalgam filling but always gold in such cases.

*Dr. Clayton*: It is the better plan to hold on to the natural covering, and to be careful not to expose the pulp, we cannot have secondary dentine and there is no covering as good as the natural one.

*Dr. Reid*: I have almost always had trouble when I used oxide of zinc and creosote, where there is no exposure I frequently use the chloride of zinc, but in cases where I find it gives trouble I get over it in this way: Simply take a solution of gutta-percha and flow it over the cavity, then mix the chloride of zinc in solution and apply it in only small quantities at a time, giving it time to harden, and then flow more over it. By applying this solution in small quantities, I think there is sufficient time for expansion while the substance is getting hardened, and I think the tooth accommodates itself to it, at any rate I experience less trouble by this way of proceeding.

*Dr. Hacker*: I had a case; it was a superior molar, in excavating I found I had wounded the nerve and it was bleeding freely, I applied some carbolic acid and stopped the hemorrhage and filled the tooth immediately, though it has been ten years since, the tooth is now seemingly in as good condition as it was at first, I find when I complete an operation at one sitting it is attended with better success.

*Dr. J. Taft*: The application of carbolic acid paste serves a purpose exactly as a scab over the surface of a wound. The application aids in closing the wound and recovery takes place. I am ready to indorse the method of prompt attention to treating and filling teeth, I think the more promptly the work is done ordinarily, the better is the result.

Adjourned till Wednesday Morning, March 3rd, 1880.

### MORNING SESSION.

### WEDNESDAY, MARCH 3rd, 1880.

The meeting being called to order by the President, Dr. Cassidy, the discussion of the second question: "Professional Culture, Scientific and Esthetic" was begun.

*Dr. J. Taft*: I suppose two distinct subjects are embraced in this question, the one, what is Scientific Culture; the other, what is Esthetic Culture. I suppose by scientific culture is meant the developing and fixing in the mind principles, laws and knowledge, so that they may serve as a basis for the practicable part of the profession, scientific culture includes the chief things that shall prepare one for practice; it is a foundation upon which to build a practical profession. We understand what is ordinarily meant by the professions, though this term is now somewhat expanded; formerly it included only theology, law and medicine, now, it is for the most part applied to those departments of human occupation, but it is being applied also to other things, more and more as the world develops, new things come up and assume the name of a profession, for example: within the last 40 or 50 years we are wont to speak of the dental profession. Now, there is a scientific part of culture that must be obtained before one can fairly enter the practical profession. The dentist has to deal with organs and tissues, many of which are very delicate. It is important for us to know as much as possible; the more light we have upon any subject, the better able will we be to go forward and accomplish our work, and this is true of dentistry as of law or medicine. In dentistry, certain kinds of knowledge are necessary, for instance, Anatomy, Physiology, Pathology, Histology, Chemistry and Therapeutics, the science that teaches us what agents to use in the restoration of diseased parts, these are some of the branches that it is necessary to understand, in order that we may have the ability to accomplish our work properly. But, no one can profitably take up these studies without preliminary preparation. Can one master Anatomy without previous preparation for its study? The question may arise, what is necessary? For the study of Anatomy, we should have some knowledge of Latin and Greek, for the nomenclature of this branch is not in the language which we every day use. The names pertaining to nearly all the parts are not anglo-saxon words; they are not words that are used in daily life or taught in the common schools; they are names mostly from Greek and Latin. If one attempts the study of Anatomy without some knowledge of these, he will find that it is chiefly a matter of memory, he will have to commit to memory names the definition of which he knows nothing. Every one is placed in this position who attempts to study Anatomy without understanding something of these languages, we all know what a difficult matter it is to commit to memory a word without knowing its meaning, when one is familiar with the meaning and derivation of a word it is far more easily retained in the memory, where it is a work of the memory without the understanding the difficulty of study is greatly increased, it is simply work of the memory without an idea, and it will soon pass away, such a one is at a disadvantage compared with one who knows the meaning of the words used; he will be saved great time and labor, for a great deal of time is consumed in memorizing. Then, the student either dental or medical, with the proper preparatory knowledge will accomplish the work more efficiently and more to the satisfaction of his teachers and himself. All this has not been properly recognized, but the time is coming when there will be a fuller appreciation of its truth. Our own language should be well mastered and the student should have a knowledge of the languages out of which our own has grown. A number of persons enter the profession ignorant of their own tongue, take any class, and so far as I have observed a large number are defective in the English language. I have found on the other hand, that those who were familiar with Greek and Latin had a decided advantage over those who had no knowledge of those languages, the science of numbers should be better understood in all its parts.. To advance properly and rapidly in any work, it is necessary to have a thorough preparation; it will assist him to make his work more efficient and greatly increase his power.

The habit of study is an important element of success. One great embarrassment in teaching is want of ability for concentration and application, it sometimes requires a whole session to bring a man down to the habit of study.

The manner in which we apply the nomenclature in our profession is an illustration of a loose study of habit, the employment of words and terms in a vague manner is very palpable in the dental profession, we use one word sometimes to mean two or three different things, and often use terms that should not be used at all, all this grows out of a lack of culture in this respect. We speak of the nerve of a tooth, and a more inapt use of a term could hardly be imagined. The pulp of a tooth is made up of connective tissue, and contains a large amount of plasm and fluid and soft solid material; in fact, a very small proportion of the pulp of a tooth is nerve, it has only had that appellation because it is possessed of exceeding sensitiveness. There is not half as much propriety in calling the pulp a nerve as there would be in calling it the plasm of a tooth, pulp is the word now used, and it is the most feasible word, but if a better word can be devised, I am ready at once to accept it. Again, we say the six year molars, or six year old molars, this is very awkward, why could we not say first permanent molar? You might as well say the eleven year old bicuspid and the twelve year cuspid. It is very easy to say first permanent molar, and every body will know what is meant, and besides it makes the nomenclature uniform, the misuse of terms shows a defective primary education. We are faulty in this respect and should seek correction as soon as possible. In regard to this matter, I think the Dental Colleges are at fault for not taking a high ground on the subject. A better course of study is necessary.

A great many boys enter dental offices as errand or labratory boys. They develop somewhat and are quick and energetic, and soon begin to ask if it would not be a good plan to study dentistry, then, after awhile they think they had better go into the college, they go to college and under great embarrassments they go through. How they succeed it hard to see, some of them possess an aptness and an ability to press themselves along wherever they may happen to be placed. They force their way through the college and the fact is some of them have little or no qualifications whatever for entering the profession, and it is too often true that the dentist from whose office they have come has encouraged them, all along, knowing their lack of qualification for the profession.

Dr. Taft here read some resolutions recently passed at a meeting of the Pennsylvania Association and commented on them. We are faulty and limited in our system of special education, the medical profession is also at fault. perhaps more so than we are, for not having a higher standard of education, but I think there will be an important advance in the matter before many years.

*Dr. Taylor:* In looking over the whole field I can see some of the difficulties that surround this subject. I have been long enough in the profession to know that a dentist cannot know too much. There is no knowledge that cannot be used at some time. Primary education has been neglected. Circumstances have drawn men into the profession who have not the least adaptability for it. But I think we are somewhat out of the old rut, for it used to be that a man who had worked three months in a silversmith's shop, and could work in gold a little was fitted to be a dentist. Now it is not so, whether it is better to raise the standard and keep those defective in literary attainments out of the college I can not tell, some of our best dentists have come in that way. A man ought to be thorough in the English branches, and you may say that is a low standpoint, and yet out of the 500 students this winter in Cincinnati in the medical schools, not two hundred of them have passed through any literary school whatever, and two-thirds of them cannot write a thesis or spell the majority of medical terms correctly. I don't believe dentistry is behind medicine, there are students entering both professions defective in literary attainments. I suppose you all feel as I do in this matter. I have been 40 years in the profession and to day I feel as if I do not know half what I should, I would go on till I knew all about galvanism and mechanism and chemistry and then I don't believe I would stop till I had made a perfect tooth, although, I don't think I would try to put a nerve in it, as some have suggested and think they can.

We must gradually come out and require a higher standard of preparation. There ought to be some examination made of applicants before they enter the college at all. The young members of the profession must recollect, that as far as they push ahead and as high as they raise the standard, just that high it will go; and I think as we have advanced in the past we will continue to advance. When we first organized we hardly knew the wants of the profession; the majority of the profession did not want anything. If we continue, it seems to me that the colleges will advance as they have done, and as the needs of the profession indicate.

*Dr. Taft*: I don't know whether it is safe to take the position that the colleges will advance as far and as rapidly as the profession demand.

*Dr. Taylor*: I will correct it by saying the colleges will advance as far as the profession is qualified.

*Dr. Taft*; It is in the teaching that we should take as high a position as the profession would permit. The colleges have fallen short. I doubt not the profession would sustain them in taking a higher position than they have; they might make a very decided advance and still be sustained by the profession. This should be done rather than to go back to see where we once stood. The progress made is of course a source of gratification, but the colleges might have gone still farther and more might have been done. The Institution now being organized in San Francisco is taking a high stand and the one in Pennsylvania and also the one connected with Harvard University, and if these can afford to do so, I am sure the older ones certainly should come up and take a high stand.

*Dr. Jay*: I think the trouble is if the standard is raised it will keep a number of students out. I have conversed with three of my friends in the medical colleges on the subject and they say it is the same; if only certain students were admitted the quality will be better but there will not be so many students to support the colleges.

*Dr. Berry*: The institutions must have funds to support the professors. The medical colleges try to crowd as many in as possible, and we must do the same; it is the necessity of the age. The colleges must have support. The students that are ambitious will rise and improve anyhow, and the money of the numbers must be had to support the schools. If you put up the standard and require the men to study reading, writing and put them along in arithmetic to say nothing of giving them a smattering of Greek, Latin, Chemistry and Anatomy, you cut off the number of students and the support of the schools.

*Dr. Howe*: The proper way it seems, would be, to begin with the local teachers. The students when entering the offices should be required to pass an examination. If the dentists would refuse to receive students in their offices unless they could pass a certain examination it might make a difference.

*Dr. Taft*: There is one branch of the topic that has not been touched. We have mainly talked of those who are about to enter the profession, but a word might be said of the progressive attainments of those already in the profession. Many imagine they have finished their education when they have passed through college, but in reality it has only begun; they are then only ready to begin the practical work. How many here can say they were satisfactorily prepared for the profession when they left the college? Very few I think, when a member who has been in the profession 40 years says he does not know half he should like to know. Many students think, they have only to open an office and wait for business when they have passed through college, think there is nothing more to be done; but this is wrong, for every spare hour should be employed in assiduously studying, and if we do not continue to progress as we go on in life, we are false to ourselves and our patients. In the study of Chemistry, Physiology, Anatomy and Pathology there is vast scope for labor; and there are none of us however old we may be but can learn something. We talk about the colleges coming up to the point required by the profession, yet after all, the strongest call for a high stand comes up from suffering humanity. They plead with us to save their teeth from the ravages of disease. We should always consider how we may best serve the interests of our patients, and bring our knowledge up to the highest point of excellence.

*Dr. Taylor*: There is a great deal in this subject. A person in the dental profession can never afford to leave off study. It seems to me a good plan would be to take a certain branch at a time and make it a special study; finish it, then take up another and in this way all spare time could be utilized. There would be Anatomy, Physiology, then the microscope might be taken up and the mucous membrane of the mouth could be better understood, and more would be known about the pulp and sensitive dentine. In a course of 40 years study, if he lives so long, a member of the dental profession would make great progress.

*Dr. Taft*: When a man has undergone a course of study he should be capable of taking up more than one subject at a time. He must have at all times a microscope, for it is almost a daily necessity; he has his attention constantly turned to Chemistry and Histology and Pathology, and a microscope is in constant demand. Mechanical appliances should always be at hand.

*Dr. Taylor*: I meant to say the student should have one principal study, and I would impress upon the young man that it is necessary to master every subject before it is laid aside.

*Dr. Taft*; There is something more than scientific attainments, I take it. This expression is a little elastic and may be allowed to cover more than mere scientific and practical attainments. I regard the quality of honesty as very important. Every dentist should be honest in his profession. All he does, he should do from pure motives from the sense of doing right. Very many persons do not know just what ought to be done; indeed very few dentists know what should be done in their own cases, and rarely will a physician prescribe for his own sickness. Dentists are left to decide for their patients and they should always act conscientiously in the matter; when the work is done he is to judge of its worth, for the patient knows but little about it. So I regard it as essential that a dentist to practice in his profession properly should be full of honest integrity and uprightness of mind. I know that many times the operator has to labor under embarrassments, that only he can appreciate, but I have many times seen work that was faulty from the beginning and willfully faulty. The dentist should make himself as acceptable to the patient as possible. I have often heard persons say of a dentist, he did this or that, he was untidy, unkempt, or his breath was foul. All these things should be garded against. And why? To secure more business? No, but because it is far better for the operator, and he can do the work more satisfactorily if his patient is at ease, if all the surroundings are pleasant. If a patient is irritated by anything, he will be likely to manifest it by becoming nervous and excited, and the operator will then have to labor under difficulties, and cannot do his work so well as if things were agreeable and the patient quiet. The operator should always adapt himself to the condition of the patient and the frame of mind he may be in. Some persons are reserved in manner and will not care to converse and others will be very talkative. The dentist should always make it a point to act in a manner pleasing to his patients. If a person does not want to talk, then the dentist should not insist on keeping up a conversation. The best thing, is to be governed by circumstances, and place the patient at his ease in all respects.

Outside of his office the dentist should deport himself in a becoming manner. He should never presume socially on his professional acquaintance. A person may come to his office for services because he thinks he can serve him well, and that person may not want to recognize and speak to him elsewhere. We do not presume to thrust our profession even on our best friends. There are persons, who will receive your advances no matter where or how you have become acquainted, but, there are others who will not care to meet you socially. It is lowering the profession when a dentist presumes too far, and if it is not acceptable to a person, you can see how much a dentist lets himself down. When we meet our brethren in the profession, we should meet as professional men, and be able to cope with them on all subjects within the scope of our professional education.

*Dr. Keely* indorsed all that had been said on the subject and spoke of how unbecoming to the profession it was for one member of it to attempt to cry down the work of another. He thought it would be well if all young dentists would cultivate a high standard of honesty and integrity in this respect, and always make their own work of a superior quality, it would be well.

*Dr. Sage*: I am here to take up the gauntlet in behalf of the young men in the profession. I wish something could be done to bring the old men out of their incompetency. My observation shows me that ignorance is displayed by the older men in the profession, and gives indication that there could be an improvement.

Meeting adjourned to meet at 2 P. M.

(TO BE CONTINUED.)

